# Comprehensive Raman study of epitaxial silicene-related phases on Ag(111)

**DOI:** 10.3762/bjnano.8.137

**Published:** 2017-07-03

**Authors:** Dmytro Solonenko, Ovidiu D Gordan, Guy Le Lay, Dietrich R T Zahn, Patrick Vogt

**Affiliations:** 1 Semiconductor Physics, Chemnitz University of Technology, 09107 Chemnitz, Germany; 2 Aix-Marseille Université, CNRS, 13397 Marseille Cedex, France

**Keywords:** epitaxial silicene, in situ Raman spectroscopy, phase diagram

## Abstract

The investigation of the vibrational properties of epitaxial silicene and two-dimensional (2D) Si structures on the silver(111) surface aims for a better understanding of the structural differences and of the simplification of the seemingly complex phase diagrams reported over the last years. The spectral signatures of the main silicene phases epitaxially grown on Ag(111) were obtained using in situ Raman spectroscopy. Due to the obvious 2D nature of various epitaxial silicene structures, their fingerprints consist of similar sets of Raman modes. The reduced phase diagram also includes other Si phases, such as amorphous and crystalline silicon, which emerge on the Ag surface at low and high preparation temperatures, respectively. The Raman signatures obtained along with their interpretations provide the referential basis for further studies and for potential applications of epitaxial silicene.

## Introduction

Epitaxial silicene, an elemental 2D silicon allotrope [1–3] grown on a supporting substrate such as Ag(111), has attracted considerable interest since its first discovery in 2012 [4–6]. Yet, the investigation of epitaxial silicene on Ag(111) remains challenging because of the complex phase diagram upon the formation of Si structures on Ag(111). It was shown that different substrate temperatures during Si deposition result in the formation of various 2D Si phases [7–8] with (3×3)/(4×4) and 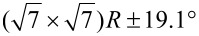/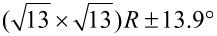 symmetry, where the first part refers to the translational symmetry of the structure with respect to silicene and the second part refers to the translational symmetry with respect to the Ag(111)-1×1 surface, and a so-called “
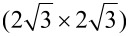
” superstructure. The angle given outside the parenthesis describes the rotational mismatch between the superstructure and the crystallographic directions of the silver substrate. Despite the clear assignment of these 2D Si layers to different symmetries, their properties and assignment to silicene are controversially discussed in the literature [9–11].

The most extensively investigated structure is the prototypical (3×3)/(4×4) phase, which is so far the only one clearly shown to refer to epitaxial silicene [4,12–13]. The 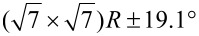/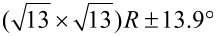 subsidiary phase can be found in four different domains [14]. These domains are explained by four different rotation angles relative to the Ag[110] direction of an initial honeycomb lattice similar to the (3×3)/(4×4) phase but slightly expanded. Because of the interaction with the Ag(111) substrate, those domains have a very different appearance in STM imaging. The 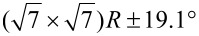/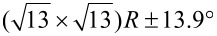 phase always coexists with the (3×3)/(4×4) and the “
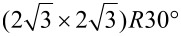
” superstructure and forms relatively small domains. Its similarity to the honeycomb (3×3)/(4×4) phase has not been proven unambiguously yet. At growth temperatures above 250 °C, the “
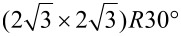
” superstructure is predominantly formed; it is the most controversially discussed Si structure on Ag(111). While Jamgotchian et al. [15] assigned this structure to a perfectly ordered silicene phase with an enhanced crystallinity if grown at 390 °C, instead, Liu et al. [16] showed that it comprises ordered and disordered areas, while Acun et al. [17] underlined the beginning of the distortion of epitaxial silicene, which leads to its destruction at 300 °C, caused by a dewetting process. Angle-resolved photoemission spectroscopy measurements of this superstructure by Wang et al. [18] showed that its electronic band structure mostly comprises bands pointing to an sp^3^ hybridization of its Si atoms. Moreover, there are also claims that this superstructure is stabilized by Ag atoms, found either on the top or inside the 2D layer [19–20] and, therefore, it would not be a real silicene phase.

In order to elucidate the complex formation of the diverse 2D Si structures on Ag(111) and to probe the nature of the silicene-related ones, we have employed Raman spectroscopy, a versatile and non-destructive optical method, highly sensitive to the structural properties of the materials [21–22]. The first results on 2D Si structures, obtained using in situ Raman spectroscopy, conclusively confirmed the 2D nature of epitaxial (3×3)/(4×4) silicene and demonstrated an easy access to its chemical and physical properties [23]. Based on these results, the spectral signatures of silicene-related superstructures are established. Our results reveal a fundamental difference among these superstructures, related to the ratio of structural order and disorder. Furthermore, the in situ Raman results allow the phase diagram to be determined for the silicon deposition onto the Ag(111) surface from room temperature (RT) up to 500 °C.

## Results

### Scanning tunneling microscopy

**Figure 1:**
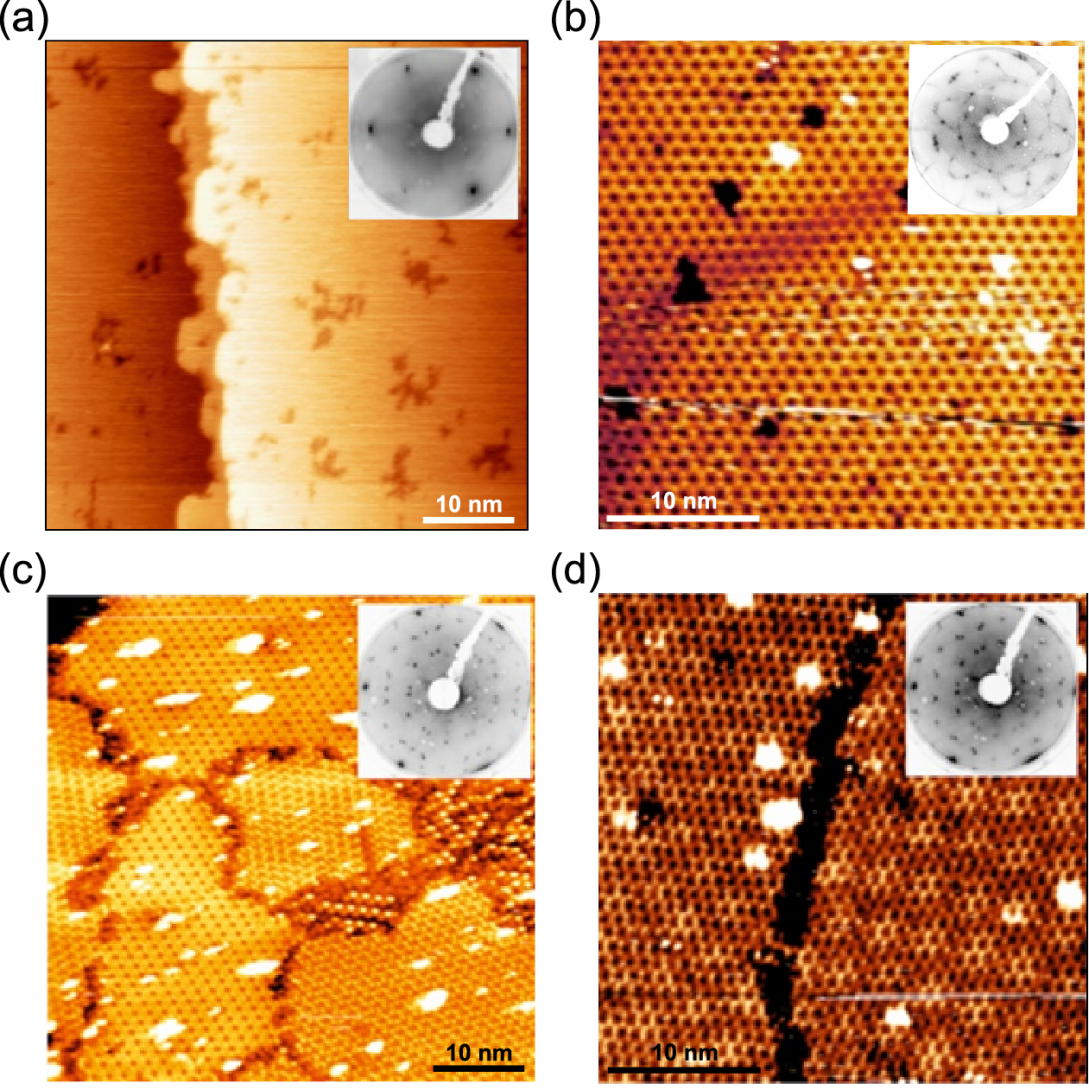
(a) STM topographic images (*U*_bias_ = −1.0 V, *I* = 1.08 nA) and corresponding low-energy electron diffraction (LEED) patterns (insets) of (a) 0.1 ML of Si deposited onto Ag(111) at room temperature, (b) 1 ML of Si deposited at 220 °C resulting in (3×3) silicene formation, (c) 1 ML of Si deposited at 240 °C showing the formation of several phases, and (d) 1 ML of Si deposited at 280 °C with a clear “
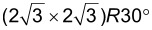
” reconstruction.

Figure 1 shows the STM images for Si deposited onto Ag(111) at different substrate temperatures in agreement with previous reports [4,8]. For deposition of about 0.1 of a ML at room temperature filled-states STM images (Figure 1a) show the formation of cluster-like structures on the otherwise atomically flat Ag(111) surface. The number and sizes of the clusters increase with Si deposition time but do not show any additional corrugation, which would be indicative for any order within the clusters. This is in agreement with the LEED observation, which shows no additional diffraction spots besides the integer-order ones of Ag(111)-1×1 even for the deposition of a complete ML. The formation of these clusters takes place up to a preparation temperature of around 170 °C. At temperatures between 180 and 210 °C the Ag terraces start to show some decoration by locally ordered features developing from the Ag step edges into the terraces (not shown). Still, no long-range order is observed. For deposition temperatures of approximately 220 °C a very clear new symmetry of (4×4) with respect to the original Ag(111) one can be seen by LEED (Figure 1b, inset). It indicates the formation of the (3×3)/(4×4) epitaxial silicene structure, shown in the STM image in Figure 1b.

At temperatures above 220 but below 250 °C the formation of multiple phases including (3×3)/(4×4), 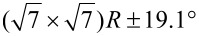/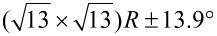 (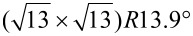, in short) and domains of a third 2D Si configuration, the “
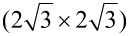
”, are found by using STM (Figure 1c). The related LEED patterns show a superposition of these three distinct symmetries, yet a slight domination of one of the symmetries depends on the exact preparation temperatures (Figure 1c, inset). The 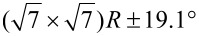/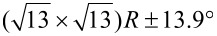 structure comprises four different domains as a result of the combination of the different rotation angles. These four domains can be clearly distinguished in STM but refer to a very similar underlying silicene honeycomb structure [14]. Contrary to the (3×3)/(4×4), it is not possible to prepare dominant multiple or single 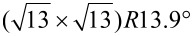 domains.

At higher preparation temperatures the “
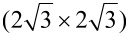
” symmetry becomes dominant in LEED measurements and is finally observed solely for deposition temperatures above 250 °C (Figure 1d, inset). The STM topography image of this debated structure (Figure 1d) reveals a Moiré-like surface pattern [6,16]. This pattern originates from locally ordered areas that are surrounded by distorted or disordered zones. The ordered areas appear brighter in filled-states STM images, thus mimicking a Moiré pattern. Because of the inherent intrinsic disorder (ID) it is not reasonable to assign any silicene symmetry to this structure, which will be referred to as “
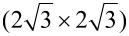
”. At higher temperatures, around 300 °C, the “
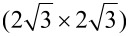
” structure finally disappears because of a dewetting process [17].

### Raman spectroscopy

**Figure 2:**
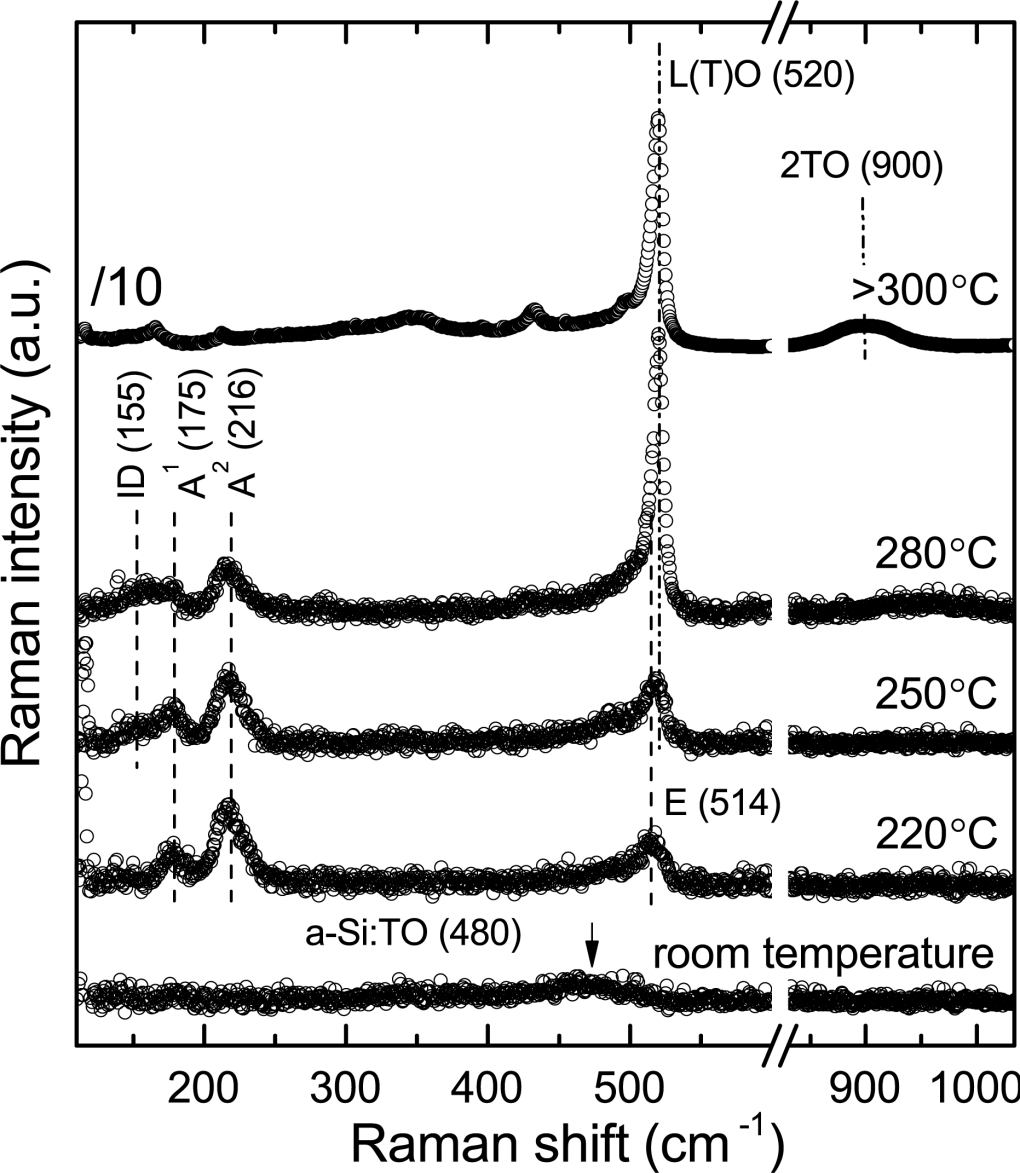
Raman spectra of the various structures obtained upon Si deposition at room temperature RT, 220, 250, 280, and 300 °C. In the spectral range between 550 and 830 cm^−1^, no features were observed. The spectra are stacked for clarity. The topmost Raman spectrum was divided by 10 to fit the rest of spectra in the plot.

Figure 2 shows the Raman spectra, obtained after Si deposition onto Ag(111) at different growth temperatures related to the different aforementioned structures. Firstly, we notice that these Raman spectra differ significantly, pointing to fundamental structural differences between the diverse growth conditions. For the growth at room temperature, the spectra exhibit a broad Raman band at 480 cm^−1^ with a shoulder around 350 cm^−1^. Narrow phonon modes indicative of crystalline order are not observed in this case. At a temperature of 220 °C at which (3×3)/(4×4) epitaxial silicene is formed, the prototypical phase with C_6_*_v_* symmetry is identified by the presence of narrow A^1^ and A^2^ modes at 175 and 216 cm^−1^, respectively, and by an E mode at 514 cm^−1^. The detailed description of the Raman signature and vibrational properties of epitaxial (3×3)/(4×4) silicene can be found in [23].

The Raman spectra of the “mixed phase” (grown at temperatures between 220 and 280 °C) exhibit similar features as those of (3×3)/(4×4) epitaxial silicene, but show also several deviations: The E mode at 514 cm^−1^ seemingly shifts to 518 cm^−1^, and a new shoulder appears at 155 cm^−1^. For even higher deposition temperatures around 280 °C the Raman spectra are dominated by a mode at 520 cm^−1^, accompanied by a decrease of all modes observed at lower deposition temperatures with the exception of the mode at 155 cm^−1^, also present in this growth regime. Its energy and line shape indicate the disorder-related nature of this Raman band, particularly evident in the case of the “
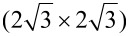
” structure, known to be mostly disordered [16]. If the growth temperature further increases above 300 °C, the related Raman spectra become dominated by an intense mode at 520 cm^−1^ and a broad band at 900 cm^−1^, showing a strong similarity to bulk diamond-like silicon. The low-intensity Raman bands below the band at 520 cm^−1^ will be discussed elsewhere.

Our Raman results differ significantly from recently published ex situ and in situ Raman observations. Previous ex situ Raman results showed the presence of an “E_2_*_g_*” mode at 516 cm^−1^ for the (3×3)/(4×4) phase, whereas the Raman spectrum of the “
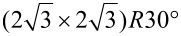
” structure was reported to exhibit strong bands at 521 and 900 cm^−1^, interpreted as a graphene-like behaviour [24]. The in situ Raman results of epitaxial (3×3)/(4×4) silicene at one monolayer coverage from Zhuang et al. [25] show bands at 230 and 530 cm^−1^, while in the work of Diaz Alvarez et al. [26] vibrational modes of the same structure are reported at 246 and 518.7 cm^−1^. In the latter case, the Raman peak at 246 cm^−1^ is found at low silicene coverages. In none of the cases the spectral signature of the accompanying 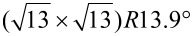 structures could be distinguished from that of the dominant (3×3)/(4×4) phases.

In order to elucidate the formation of the different Si phases on Ag(111) and their related properties we now look at the Raman signatures of these phases (shown in Figure 2) and compare them to the signatures of (3×3)/(4×4) epitaxial silicene. We start with the lowest (*<*170 °C) and the highest (≥300 °C) growth temperatures, which also mark the 3D transition for the 2D structures on Ag(111).

### Si deposition at room temperature

**Figure 3:**
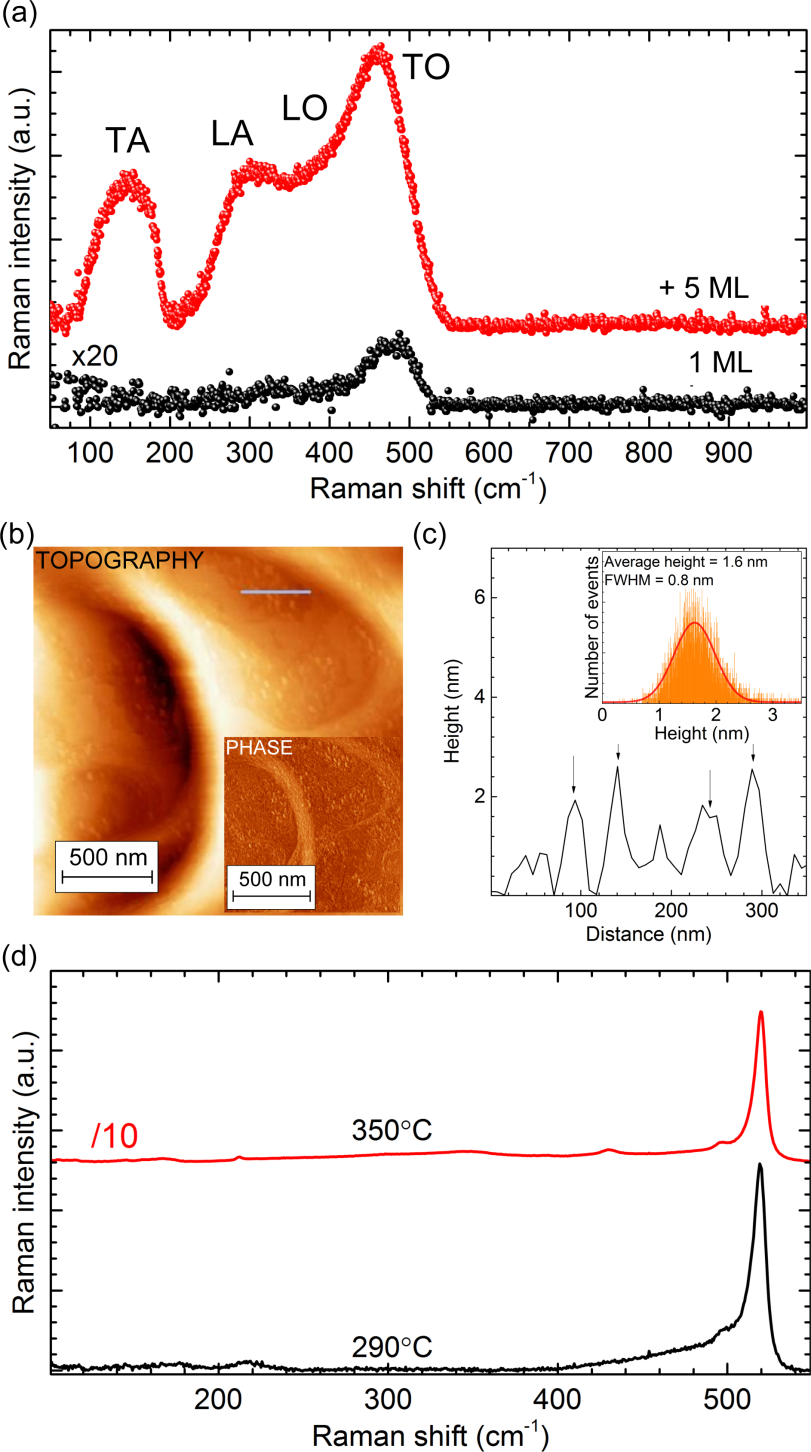
(a) Raman spectra recorded on samples after Si deposition at room temperature with coverages of 1 ML and 5 MLs. (b) Ex situ AFM topography measurement of the Ag(111) surface after the 1 ML deposition at room temperature. Inset: phase image. (c) Height profile, along the line in (b). Inset: height distribution of the features in the AFM topography image. (d) Raman spectra of samples with nominal 1 ML amount of Si deposited at 290 °C and 350 °C.

A detailed Raman spectrum recorded on a sample after the deposition of nominally 1 ML of Si at room temperature is shown in Figure 3a together with a sample prepared under identical conditions but with 5 MLs of Si deposited. It is clearly seen that the sample with 5 MLs of Si shows similar broad bands at 350 and 480 cm^−1^ when compared to the sample with 1 ML of Si but the shoulder at 350 cm^−1^ and a less intense broad band at 150 cm^−1^ are more pronounced. The shoulder at 350 cm^−1^ is actually a combination of several Raman bands located around 310 and 380 cm^−1^. This spectrum shows strong similarity to the one of amorphous Si, which is characterized by comparable Raman bands [27–28]. For the deposition of 1 ML the Raman intensity of all bands is very weak: Practically, only the most intense mode at 480 cm^−1^ is detected.

Ex situ AFM images of this sample recorded under ambient conditions, i.e., after oxidation in air, are displayed in Figure 3b. Raman spectra obtained before and after air exposure are identical confirming that the oxidation process in air does not cause the appearance of those new features. Numerous bright features having an average height of about 2 nm (Figure 3c) can be found now on the surface within the scanning range. The contrast in the AFM phase image in the inset of Figure 3b demonstrates the different chemical compositions of the bright features and of the Ag(111) surface. In combination with the Raman results we conclude that these small structures are related to amorphous silicon (a-Si). This demonstrates that the Si deposition at low temperatures (≤150 °C) produces neither ordered 2D nor 3D crystalline Si structures. We can assume that the Si deposition at even lower temperatures (≤20 °C) leads to a similar result. It was recently suggested that impinging Si atoms at room temperature penetrate the Ag(111) surface, exchange with Ag atoms and act as seeds for the growth of recessed islands [29]. At the same time the released Ag atoms would form new Ag(111) terraces by a process described to occur more rapidly as the size of the embedded islands increases. These assumptions are not supported by our results, which demonstrate that room-temperature deposition only leads to the formation of amorphous Si clusters.

### Si deposition at temperatures above 300 °C

If Si is deposited onto Ag(111) at substrate temperatures exceeding 300 °C, only the characteristic (1×1) pattern of the initial Ag 1×1 surface is found without any distinctive additional diffraction spots.

Figure 3d shows the Raman spectra after deposition of 1 ML of Si at 290 and 350°C, both dominated by an intense band at 520 cm^−1^ with a FWHM of 8 cm^−1^. This mode is similar to the L(T)O phonon mode of diamond-like silicon, clearly indicating the formation of Si crystallites. Additionally, the second-order TO phonon mode around 900 cm^−1^ (Figure 2, top spectrum) supports the bulk-like nature of the structures formed. The fact that the intensity of the L(T)O phonon mode gets higher for deposition at 350 °C demonstrates that the sizes of the crystallites enlarge with increasing deposition temperatures. However, this temperature is still low compared to the growth temperature of crystalline Si, which usually exceeds 1000 °C [30]. Such a low crystallization temperature is surprising, but it can be explained by metal mediation. For a layered Si–Ag system a temperature as low as 400 °C was reported [31].

These results are in agreement with Auger electron spectroscopy measurements [16] and low-energy electron microscopy observations [17] as well as Raman results after post-annealing of the (3×3)/(4×4) epitaxial silicene phase [23], which demonstrated a dewetting process of the Si layer from the Ag(111) surface around 300 °C. Hence, a temperature of 300 °C marks the high temperature limit for 2D Si layer formation on Ag(111), where a 2D-to-3D phase transition takes place. Reports of an almost perfectly ordered 2D Si layer formed on Ag(111) at almost 400 °C may be related to problems with temperature determination [15].

### Si deposition at temperatures between 220 and 290 °C

**Figure 4:**
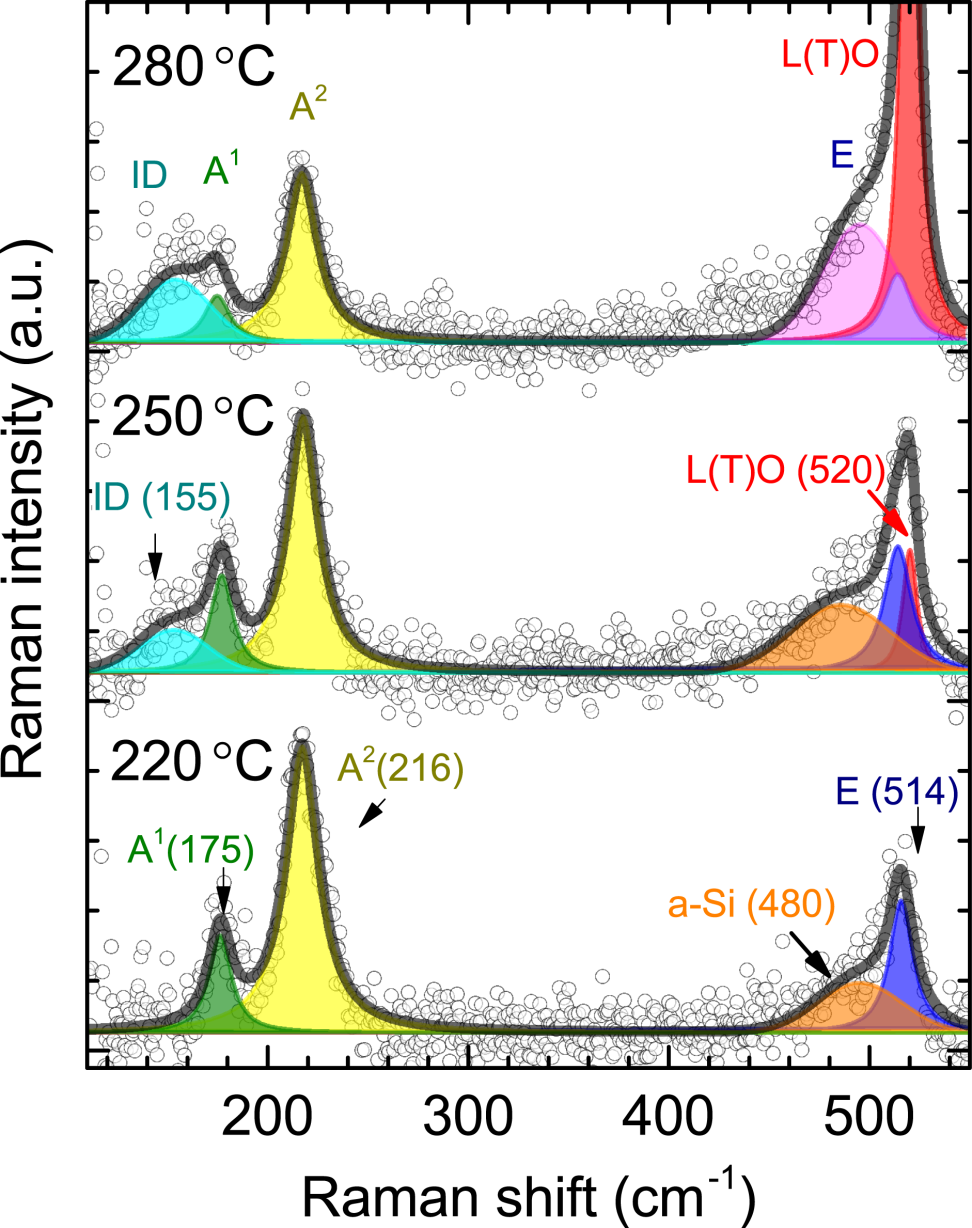
Fitted Raman spectra of silicene-related structures: dominant (3*times*3)/(4×4) (epitaxial silicene) (220 °C), “mixed phase” (250 °C), and “
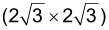
” structure (280 °C). The experimental data are shown as circles, while the smooth curves overlaid are the envelopes of all features observed and fitted. The spectral features are fitted with Voigt functions, where the Gaussian contribution of the peaks stems from the instrumental resolution (2.4 cm^−1^) unless stated otherwise.

All the results presented so far show that the formation of 2D Si layers on Ag(111) is limited to a temperature range between 220 and 290 °C. We show fitted Raman spectra of 2D Si layers prepared at three different deposition temperatures within this temperature range in Figure 4.

At 220 °C the spectrum of a dominant (3×3)/(4×4) silicene layer shows all the modes of epitaxial silicene as well as a small contribution from a-Si (orange feature). The Raman spectra after Si deposition at 250 °C referred to as “mixed phase” are composed of six modes. At a deposition temperature of 280 °C a single “
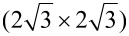
” structure is observed. We first focus on the “mixed phase”.

**Figure 5:**
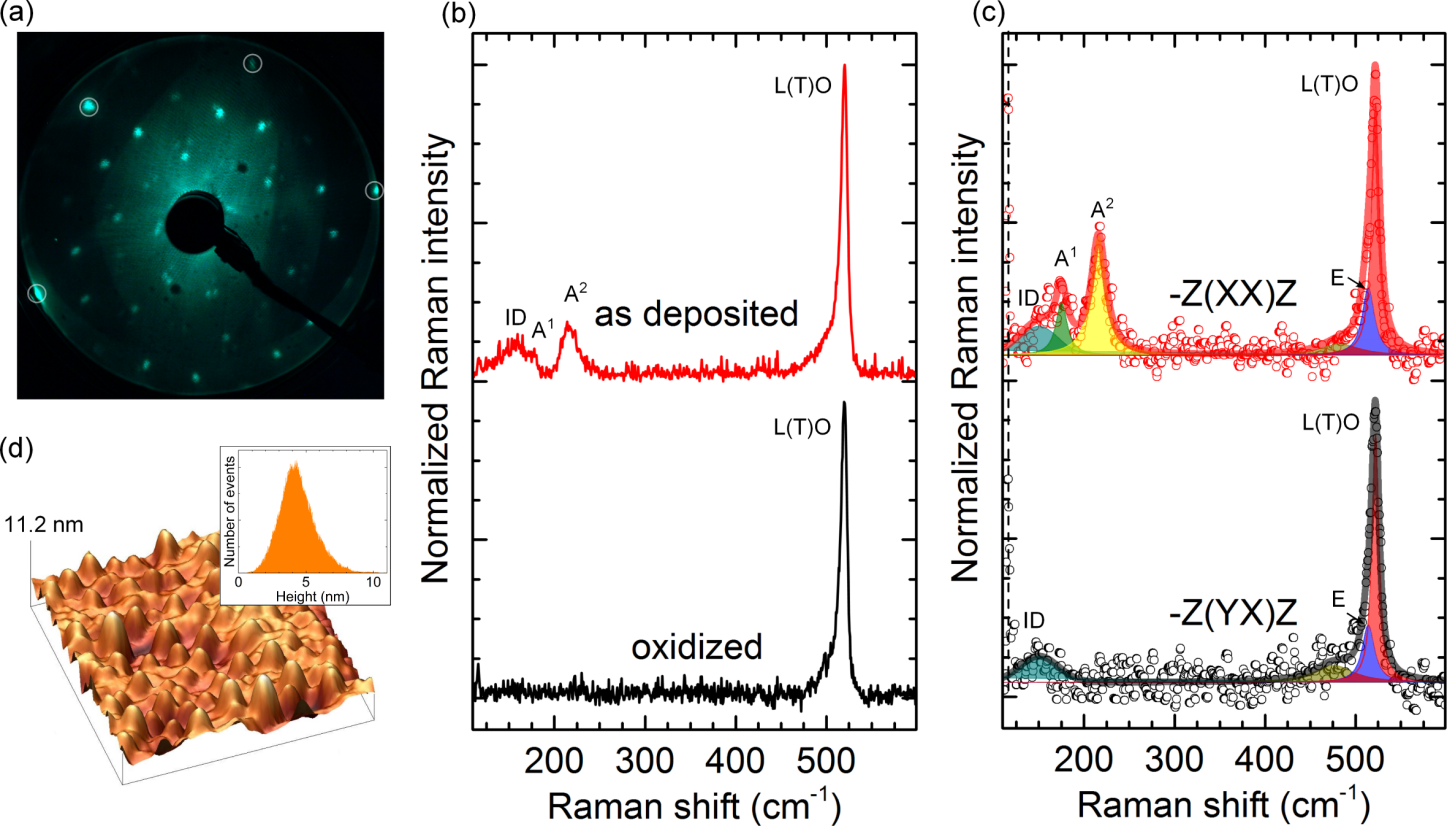
(a) LEED pattern of the sample prepared at 280 °C. The integer-order diffraction spots of Ag(111) are marked with circles. (b) Raman spectra of the sample with the “
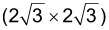
” structure after deposition (corresponding to panel a) and after ex situ oxidation. (c) Polarization-dependent Raman spectra of the 280 °C sample, recorded in parallel (−*z*(*xx*)*z*) and crossed (−*z*(*yx*)*z*) geometries. (d) Ex situ AFM image (1 μm × 1 μm) of the Ag(111) surface, measured after the oxidation. Inset: height distribution of the small features observed in the AFM topograph.

The spectrum of the sample prepared at 250 °C exhibits the same Raman bands as those of epitaxial silicene (Figure 4, bottom) plus two additional Raman modes at 155 and 520 cm^−1^. The latter is consistent with the position of the L(T)O phonon mode of Si crystallites clearly visualized in AFM at higher deposition temperature (300 °C). This indicates that the formation of diamond-type Si starts to take place below 300 °C. It is noteworthy that the shoulder at the lower-energy side of the L(T)O mode is assigned to the crystallites and not to amorphous Si because of its consistency with the defect-TO band of bulk silicon at 495 cm^−1^. Moreover, it remains in the Raman spectrum after oxidation of the corresponding sample (Figure 5b).

The Raman band at 155 cm^−1^, however, can be attributed to the formation of the “
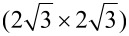
” structure, which is co-formed with epitaxial silicene in the “mixed phase”. Indeed, this Raman mode is also observed in the Raman spectrum of the sample that shows a LEED pattern assigned only to the “
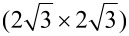
” structure (Figure 5a). In this case, the respective intensity of this mode is higher than those of the modes of epitaxial silicene. Our results of the fitting suggest that the “mixed phase” can be understood as the lateral co-existence of two different 2D structures. At the same time, it contains patches of the 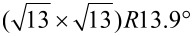 structure, which, however, cannot be distinguished from (3×3)/(4×4) silicene spectroscopically. Such spectral blending clearly suggests their structural similarity, which was also argued based on the STM results [10].

### Raman spectroscopy of the “(2√3×2√3)” structure

To elucidate the origin of the spectral signatures of the “
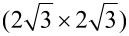
” structure, the sample was oxidized. After oxidation no LEED diffraction spots, except for the integer Ag(111) spots can be seen (Figure 5a) and the Raman modes related to the epitaxial silicene vanish (Figure 5b). In Figure 5b the Raman spectra of the “
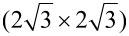
” structure before and after oxidation are shown. In the latter case the remaining Raman bands are the one at 520 cm^−1^ as well as its low-energy shoulder around 495 cm^−1^. This clearly resolves the assignment of this shoulder to Si crystallites. Ex situ AFM measurements of the same sample show protrusions with an average height of 4.4 ± 0.1 nm and a lateral size of up to 100 ± 10 nm (Figure 5d).

In combination with the Raman results after the oxidation of the “
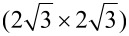
” structure, it can be stated that these protrusions are diamond-like Si crystallites. Their broad size distribution explains the linewidth of the Raman band at 520 cm^−1^: The biggest crystallites (*>*7 nm) exhibit the intense L(T)O phonon mode, while the small ones (*<*7 nm) are responsible for the large linewidth and the low-energy shoulder. The formation of such crystallites is clearly temperature-dependent. Solely 3D growth is observed when the temperature reaches 300 °C, i.e., the limit of the 2D Si-layer growth mode on Ag(111). The Raman and AFM results confirm the co-existence of Si crystallites and of the “
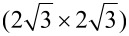
” structure at temperatures between 250 and 300 °C.

To substantiate the understanding of the “
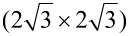
” structure polarization dependent Raman measurements were performed. Figure 5c shows Raman spectra of a sample with the “
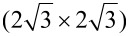
” structure in both parallel (Porto notation: −*z*(*xx*)*z* and crossed −*z*(*yx*)*z* geometries. According to the selection rules of the six-fold symmetry, the depolarized (degenerate) modes are measured in both parallel and crossed geometries, while the polarized vibrational modes can solely be detected in the parallel configuration. One notices that only A modes at 175 and 216 cm^−1^ are missing in the crossed geometry, while the Raman modes at 155, 514 (E mode), and 520 cm^−1^ remain. The polarization dependence of A and E modes fully reproduces our previous results [23]. The behaviour of the triple-degenerate Raman band at 520 cm^−1^ is also identical. The presence of symmetric modes in the Raman spectrum of the “
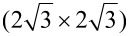
” structure at the same positions as the ones of epitaxial (3×3) silicene suggests structural similarities for these two cases. Indeed, the bright hexagons (Figure 1d) are nicely ordered and, therefore, can provide the same spectral response. We can surmise the appearance of the E mode in the asymmetric shoulder of the L(T)O phonon mode of Si nanocrystallites, yet the analysis is complicated.

The Raman band at 155 cm^−1^ is present in both geometries, which hints at its disorder-related origin, since only the vibrations of ordered crystalline structures follow Raman selection rules. Its broad linewidth of 30 cm^−1^ further corroborates this assignment. Finally, its position could be related to the softening of the A^1^ mode, in connection with the intrinsic disorder (ID) of the “
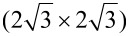
” structure. Since this Raman mode is the one that distinguishes this structure from epitaxial silicene, it can be used as a marker. Due to its evident relation to the intrinsic disorder of the “
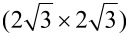
” superstructure, we refer to it as the “ID” mode. It is noteworthy that the intrinsically complex atomic arrangement of the “
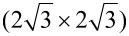
” structure shows the spectral features both of ordered and disordered nature. This has to be explicitly considered in the modeling of this structure and of its properties in DFT calculations. Proposed structural models that are entirely based on the ordered parts of this structure are genuinely bound to fail in the correct description of the “
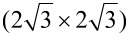
” structure.

## Discussion

**Figure 6:**
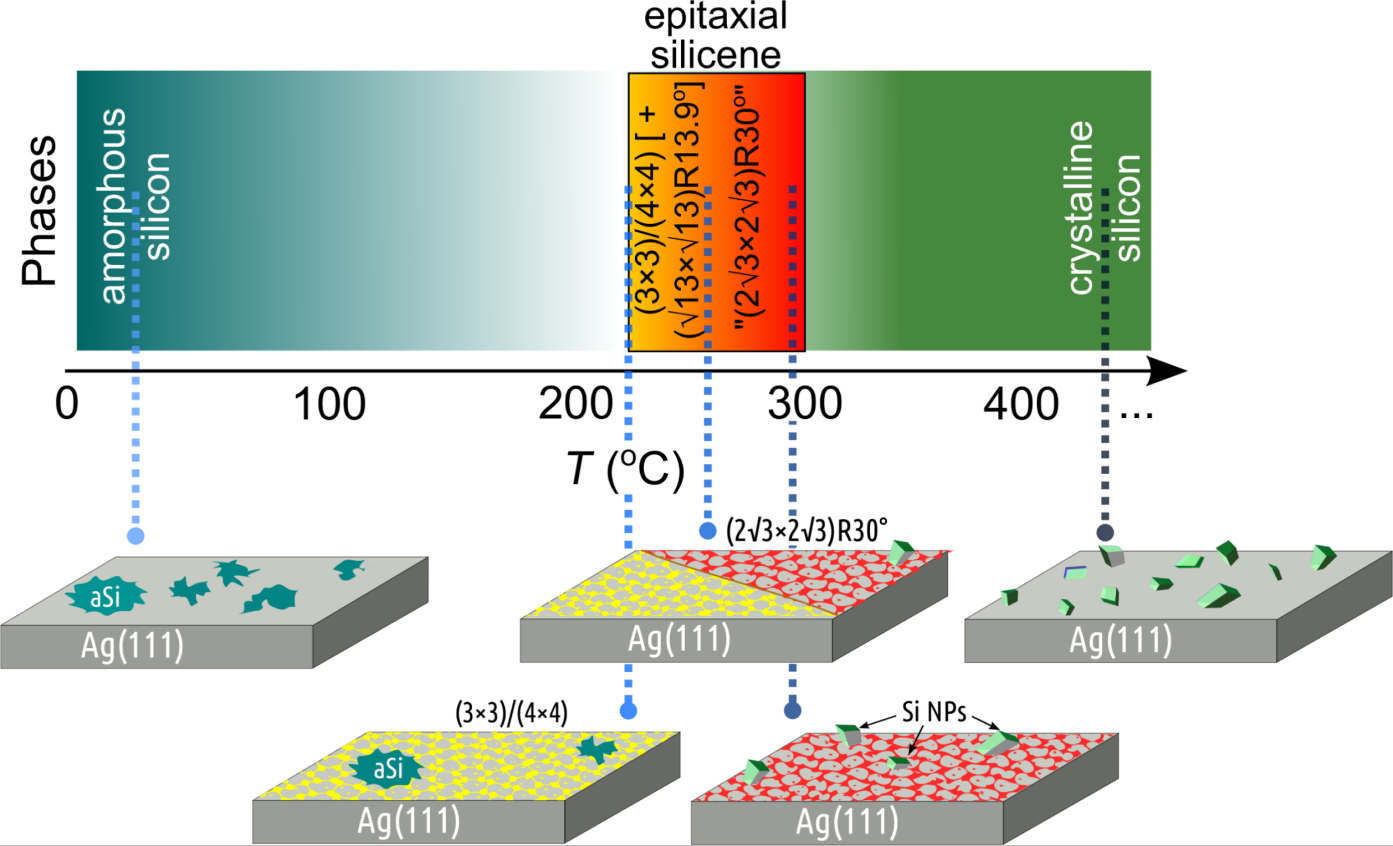
Reduced phase diagram of Si structures that can be grown on the Ag(111) surface at various deposition temperatures.

Based on the in situ Raman results in combination with STM and LEED described above, a generic phase diagram for the formation of Si structures on the Ag(111) surface can be obtained (Figure 6). At low temperatures, i.e., from room temperature to ca. 150 °C only amorphous Si is formed. In the temperature range between 200 and 300 °C 2D and 3D Si phases are formed, while at high temperatures above 300 °C only 3D Si crystallites develop.

In the narrow temperature window between 220 and 280 °C the metastable 2D Si phases are observed. Starting at 220 °C a dominant (3×3)/(4×4) silicene phase is formed; it is characterized by two vibrational A modes at 175 and 216 cm^−1^ and an E mode at 514 cm^−1^. At higher temperatures an increasing mixture of 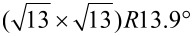 domains and around 250 °C also of a “
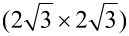
” structure are formed. All these structures show similar vibrational modes in the Raman spectra. Only the “
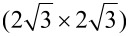
” structure shows the additional characteristic ID mode at 155 cm^−1^, which stems from its inherent disorder. For preparation temperatures around 250 °C the beginning of the co-development of diamond-like Si crystallites is observed. They further grow with increasing deposition temperatures. This scenario is in agreement with LEED observations, which show that these different structures are simultaneously observed in this temperature range. For higher preparation temperatures, the contribution of the “
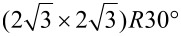
” structure increases. It is mainly described by an appearance of the ID mode at 155 cm^−1^. Accordingly, the features at 175 and 216 cm^−1^, which are dominating in the spectrum of (3×3)/(4×4) silicene, decline gradually. This means that the evolution of the Raman spectra for any multiple-phase sample can be simply explained by the weighted superposition of the Raman spectra of (3×3)/(4×4) silicene and of “
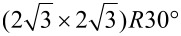
” structures.

The incidence of a Raman band around 520 cm^−1^ is a direct evidence of bulk Si crystallites present on the Ag(111) surface, but not of a 2D layer, in particular not the “
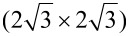
” structure, as reported earlier [24,32]. However, due to their possible co-existence, the occurrence of diamond-like Si crystallites does not exclude the presence of 2D Si layers on Ag(111).

## Conclusion

We performed a comprehensive spectroscopic study of the silicene-related superstructures epitaxially grown on the Ag(111) surface by in situ Raman spectroscopy. The structural differences between the silicene-related phases, consistent with the scanning tunnelling microscopy observations, are manifested in the sets of Raman bands, i.e., in different spectral signatures. Our results confirm a close link between epitaxial (3×3)/(4×4) silicene and the silicene-related “
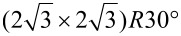
” structure since both share similar spectral fingerprints. The ordered parts of the “
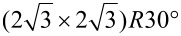
” structure exhibit a spectrum similar to that of the epitaxial (3×3/(4×4) silicene, while the disordered parts yield a broad Raman band (ID) at 155 cm^−1^. We have established that Si deposition onto the Ag(111) surface in the range from 220 to 290 °C usually results in the co-formation of 2D and 3D structures, whereas only structures with sp^3^-hybridized Si atoms are obtained outside this temperature range. Raman spectroscopy results were consistently confirmed by AFM and STM observations. According to these findings we could build up a generic phase diagram that reflects the complicated interplay of the formation of both 2D and 3D moieties.

## Experimental

Clean Ag(111) surfaces were prepared by alternating cycles of sputtering (Ar^+^, 1.5 keV, 1·10^−5^ mbar) and annealing (520 °C) until sharp 1×1 spots of the unreconstructed surface were observed by LEED. Si was evaporated subsequently from a directly heated silicon wafer piece placed at a distance of 10 cm from the Ag substrate. The Si deposition, at which a complete Si monolayer is formed, i.e., no formation of a second layer occurs, refers to “1 ML deposition”. The temperature of the Ag substrate was varied from room temperature up to 500 °C. In situ Raman measurements were performed in macro configuration, using a Dilor XY800 triple monochromator, equipped with a CCD camera as a detector. All spectra were recorded at room temperature and under ultrahigh-vacuum conditions at a base pressure of 2·10^−10^ mbar. For the excitation the 514.5 nm line of an Ar^+^ laser, with a power density below 10^3^ W/cm^2^, was used. LEED patterns were acquired in the energy range below 50 eV using a SPECTALEED, Omicron NanoScience optics.
